# The hidden genomic diversity of ciliated protists revealed by single-cell genome sequencing

**DOI:** 10.1186/s12915-021-01202-1

**Published:** 2021-12-14

**Authors:** Wenbing Chen, Changling Zuo, Chundi Wang, Tengteng Zhang, Liping Lyu, Yu Qiao, Fangqing Zhao, Miao Miao

**Affiliations:** 1 Savaid Medical School, University of Chinese Academy of Sciences, Beijing, 100049 China; 2 Beijing Institutes of Life Science, Chinese Academy of Sciences, Beijing, 100101 China; 3 Institute of Evolution & Marine Biodiversity, Ocean University of China, Qingdao, 266003 China; 4 Key Laboratory of Mariculture (OUC), Ministry of Education, Qingdao, 266003 China

**Keywords:** Ciliate, Euplotia, Genetic diversity, Single-cell genomics

## Abstract

**Background:**

Ciliated protists are a widely distributed, morphologically diverse, and genetically heterogeneous group of unicellular organisms, usually known for containing two types of nuclei: a transcribed polyploid macronucleus involved in gene expression and a silent diploid micronucleus responsible for transmission of genetic material during sexual reproduction and generation of the macronucleus. Although studies in a few species of culturable ciliated protists have revealed the highly dynamic nature of replicative and recombination events relating the micronucleus to the macronucleus, the broader understanding of the genomic diversity of ciliated protists, as well as their phylogenetic relationships and metabolic potential, has been hampered by the inability to culture numerous other species under laboratory conditions, as well as the presence of symbiotic bacteria and microalgae which provide a challenge for current sequencing technologies. Here, we optimized single-cell sequencing methods and associated data analyses, to effectively remove contamination by commensal bacteria, and generated high-quality genomes for a number of Euplotia species.

**Results:**

We obtained eight high-quality Euplotia genomes by using single-cell genome sequencing techniques. The genomes have high genomic completeness, with sizes between 68 and 125 M and gene numbers between 14K and 25K. Through comparative genomic analysis, we found that there are a large number of gene expansion events in Euplotia genomes, and these expansions are closely related to the phenotypic evolution and specific environmental adaptations of individual species. We further found four distinct subgroups in the genus Euplotes, which exhibited considerable genetic distance and relative lack of conserved genomic syntenies. Comparative genomic analyses of Uronychia and its relatives revealed significant gene expansion associated with the ciliary movement machinery, which may be related to the unique and strong swimming ability.

**Conclusions:**

We employed single-cell genomics to obtain eight ciliate genomes, characterized the underestimated genomic diversity of Euplotia, and determined the divergence time of representative species in this subclass for the first time. We also further investigated the extensive duplication events associated with speciation and environmental adaptation. This study provides a unique and valuable resource for understanding the evolutionary history and genetic diversity of ciliates.

**Supplementary Information:**

The online version contains supplementary material available at 10.1186/s12915-021-01202-1.

## Background

Ciliates, the unicellular organisms with the most complex lifestyles, are found anywhere there is water [1]. Unlike other eukaryotes, ciliates have two types of cell nuclei: silent diploid micronuclei and highly transcribed polyploid macronuclei. The micronucleus carries all the genetic material and is responsible for the transmission of genetic material during sexual reproduction, while the macronucleus genome usually has tens of thousands of copies of the genome and is responsible for the expression of genes [2]. Noncoding RNAs mediate the massive replication and recombination of the micronucleus genome and the addition of telomeres to each chromosome, eventually generating the macronucleus genome [3]. The investigation of this unique process led to the discovery of nucleases and telomeres [3]. However, this phenomenon also poses an obstacle to the study of the ciliate genome. Most ciliates feed on bacteria and microalgae [4], and some ciliates establish transient or stable symbiotic relationships with their prey [5, 6]. These cohabiting or endosymbiotic bacteria or microalgae pose a great challenge for access to the ciliate genome, especially for nonculturable ciliates.

There have been a large number of gene duplication events in ciliate genomes, bringing their gene numbers close to those of many plants and metazoans [7]. These gene duplication phenomena provide good material to study the genomic diversity and evolution of ciliates. However, due to the lack of complete genomic information, these studies have been conducted in only some model organisms, such as *Tetrahymena* [8] and *Paramecium* [7]. There are approximately 27,000–40,000 known species of ciliates [1], but genomic or transcriptomic data available for fewer than 60 species, which greatly limits the knowledge of their genomic diversity.

**Fig. 1:**
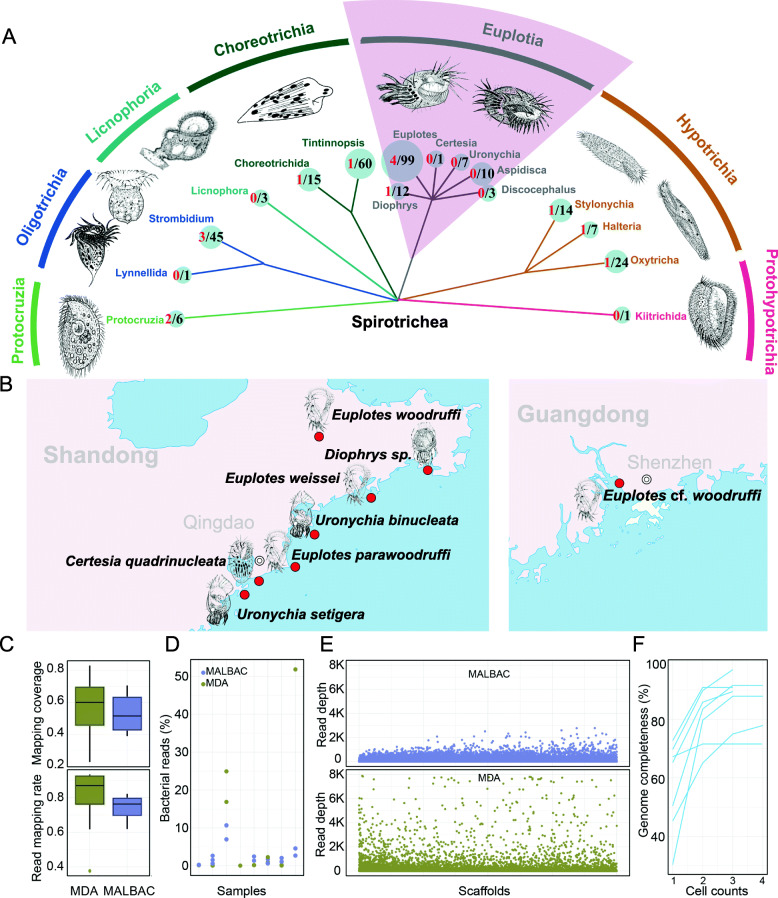
Sample collection and single-cell genome sequencing of eight species in Euplotia. **A** The taxonomy and the number of sequenced genomes or transcriptomes in Spirotricha. The size of nodes is proportional to the counts of recorded species shown in black. The number of sequenced species is shown in red. **B** The morphology and sampling sites of the eight species in Euplotia. **C** Comparison of read mapping rate and sequencing coverage between the two whole-genome amplification methods. **D** Percentage of bacteria-derived reads in each cell. **E** Read mapping depth on the assembled genome using MALBAC and MDA. **F** The correlation between genome completeness and the cell number used in single-cell genome sequencing

In recent years, single-cell sequencing technology has been applied to the sequencing of unculturable ciliates, which has helped us to solve controversial problems in ciliate phylogenetic and genomic studies [9, 10]. However, previous single-cell sequencing methods were unable to obtain complete ciliate genomes and had substantial bacterial sequence contamination, which posed a significant challenge for subsequent ciliate genome analysis. In this study, we took full advantage of different single-cell sequencing technologies and efficient genome assembly methods to obtain eight high-quality Euplotia genome sequences with low contamination. Euplotia, which contains hundreds of species, is a highly diverse subclass in Spirotricha (Fig. 1A). Through comparative genomic analyses, we revealed the genomic diversity of ciliate Euplotia, resolved their phylogenetic relationships, and systematically described their genomic features in relation to environmental adaptation.

## Results

### De novo sequencing and assembly of eight Euplotia genomes

Eight Euplotia samples were collected from coastal waters or freshwater tanks in Qingdao and Shenzhen in China. Five of them inhabit seawater, two brackish water and one freshwater (Fig. 1B). Based on morphological identification, they were classified as *Certesia quadrinucleata*, *Diophrys* sp., *Euplotes* cf. *woodruffi*, *Euplotes parawoodruffi*, *Euplotes weissei*, *Euplotes woodruffi*, *Uronychia binucleate*, and *Uronychia setigera*, respectively. These eight species represent four major genera (*Certesia*, *Diophrys*, *Euplotes*, and *Uronychia*) in the order Euplotida, whose morphological characteristics have been described previously (Fig. 1B) [4, 11–15].

To obtain clean ciliate cells and eliminate potential contamination, we picked and washed ciliate single cells multiple times using glass pipettes. Considering the difficulty of amplifying whole genomes from single cell, we used both the multiple displacement amplification (MDA) [16] and multiple annealing and looping-based amplification cycle (MALBAC) [17] approaches to amplify genomic DNA from single cells. For each method, we performed the amplification experiments twice and only one cell was used in each experiment (Additional file 1: Table S1). We constructed DNA libraries for each amplification sample and sequenced them using the Illumina NovaSeq 6000 platform. After filtering low-quality reads, the sequencing data for each cell were classified using Kraken [18], and we found MALBAC had a lower bacteria contamination (Fig. 1C, D). To remove potential contamination from endosymbiotic bacteria or microalgae, we employed a filtration pipeline (see the “Methods” section) and ultimately discarded 0.07–28% of the sequencing data. We then assembled the sequence data for each cell separately and mapped the reads back to the assemblies. MDA had a higher read mapping rate (Fig. 1C), whereas MALBAC had a lower amplification bias (Fig. 1E). After merging the filtered data for each species, we finally generated an average of 15~71 Gb data for each species with high sequencing depth (156~700×). To assess the effect of cell number on genome completeness, we combined the assemblies for each species and evaluate the genome completeness using EukCC [19]. The genome completeness significantly increased with the number of cells used until four assemblies were used (Fig. 1F).

After genome assembly and removal of contaminated contigs, we finally obtained eight ciliate macronuclear genomes with 37,973~89,285 contigs and an N50 length of 934~2,543. The genome size (74~125 Mb) and GC content (32.0%~45.9%) were similar to those of other macronuclear genomes of *Euplotes* (Table 1). To evaluate the completeness of the assembled genomes, all filtered reads were mapped back to their corresponding assemblies. Approximately 94% of reads could be aligned to the assemblies, and the remaining reads were found to be of bacterial or fungal origin. We further used EukCC [19] and BUSCO [20] to estimate the completeness of the assembled genomes (Additional file 1: Fig. S1) and found that all of them exhibited relatively high completeness ratios, ranging from 71 to 96%, which is comparable to three previously sequenced *Euplotes* cultured species: 95% for *Euplotes focardii (accession number in NCBI: GCA_001880345.1),* 95% for *Moneuplotes crassus (accession number in NCBI: GCA_001880385.1),* and 84% for *Euplotes vannus* [21] (Table 1)*.* We employed a comprehensive strategy to annotate the genome by combining homolog-based and ab initio approaches. We finally obtained 9382~25,327 protein-coding genes for each species. Most contigs contained 1~3 genes, which is consistent to previous studies (Additional file 1: Fig. S2). We found that many genes were assigned to families of closely related genes, which may be derived from gene duplication events. To determine the phylogenetic relationships of the Euplotia and confirm the morphology-based classification, we performed phylogenetic analysis using 157 orthologous genes in all available ciliate genomes using the maximum likelihood (ML) method [22]. The phylogenetic tree showed a similar topology to the phylogenetic analysis based on small subunit rRNA (SSrRNA) reported previously [23, 24]. *Uronychia* and *Diophrys* branched earliest in Euplotia and had a closer relationship to each other than to other species. *Certesia* is sister to *Euplotes*, forming a monophyletic clade that also includes *Moneuplotes crassus* (Additional file 1: Fig. S3).

**Table 1:** Summary of Euplotia genome assembly and annotation

Species	Filtered reads (Gb)	Genome size (bp)	N50 (bp)	GC content (%)	Genome completeness (%)	Gene number
***Certesia quadrinucleata***	59.6	74,018,178	1270	42.31	71.58	15,982
***Diophrys*** **sp.**	55.1	125,778,342	1838	36.04	90.91	25,327
***Euplotes*** **cf.** ***woodruffi***	51.4	83,224,465	2696	36.76	96.97	14,298
*Euplotes focardii*	/	42,117,764	1929	32.02	95.79	9382
***Euplotes parawoodruffi***	39.6	83,441,685	1610	43.02	89.47	19,635
*Euplotes vannus*	/	84,760,489	2692	36.89	84.85	15,078
***Euplotes weissei***	66.7	84,107,277	934	43.86	77.89	21,295
***Euplotes woodruffi***	53.0	87,592,728	1887	37.72	91.58	15,830
*Moneuplotes crassus*	/	51,565,225	1761	36.91	95.79	11,481
***Uronychia binucleata***	15.6	68,224,519	2316	45.86	78.79	19,733
***Uronychia setigera***	70.9	111,284,557	2543	36.43	87.88	20,152

### Euplotia is the most diverse subclass in Spirotricha

**Fig. 2.**
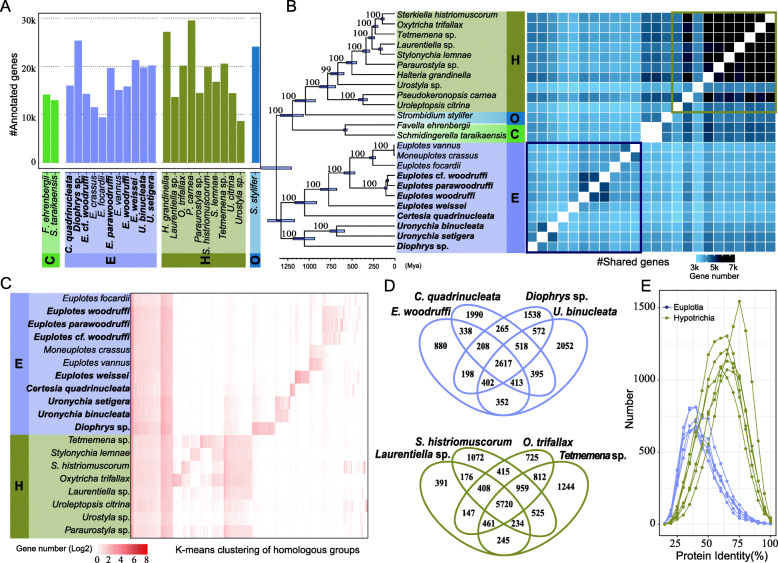
Comparative analysis of 24 ciliate genomes in Spirotricha. **A** The number of annotated genes matched to the protein nonredundant database. **B** Phylogenetic tree and heatmap of shared genes. The time tree on the left was constructed by BEAST, and the numbers on the nodes represent the bootstrap values estimated by the ML method. The heatmap on the right represents the count of shared genes among these species. **C** The k-means clustering of the homologous groups based on gene counts. Each column represents a homologous group, and each row represents a species. **D** Venn diagrams of shared homologous groups among representative species of Euplotia and Hypotrichia. **E** The distribution of protein identities among representative species of Euplotia and Hypotrichia

To compare the genome characteristics of Euplotia to their relatives in Spirotricha, we further analyzed 14 genomes and 2 transcriptomes of Spirotricha in addition to the eight genomes generated in this study (Additional file 1: Table S2). These 24 species were classified into four subclasses: Euplotia, Hypotrichia, Oligotrichia, and Choreotrichia. After genome annotation, we obtained an average of 17,634 protein-coding genes (Fig. 2A). To evaluate the divergence time of Spirotricha, we performed molecular clock analysis using BEAST [25] with the Tintinnids fossil record time, which was dated back to 580 million years ago (Mya). The time tree showed a similar topology to the ML tree described above (Additional file 1: Fig S3, Fig. 2B). Euplotia may have appeared 1300 Mya after it diverged from Choreotrichia. *Uronychia* and *Diophrys* originated from family Uronychiidae and diverged 1100 Mya. In contrast, Hypotrichia, another highly diverse subclass, originated 1000 Mya, significantly later than Euplotia (Fig. 2B). Notably, the divergence time of Euplotia at the species level was earlier than that of Hypotrichia at the genus level.

To further investigate the gene content of these 24 species, we performed homology analysis using OrthoFinder [26] and counted the homologous groups shared between each pair of species. A total of 316,565 genes were clustered into 23,294 homologous groups, of which a large majority (91.7%) could be assigned to homologous groups; the remaining 17,993 genes were species-specific. Euplotia had approximately 3805 homologous groups shared by all 11 species, significantly lower than the number in Hypotrichs (5689 homologous groups) (Fig. 2B). We further obtained single-copy genes through pairwise comparison and performed phylogenetic analysis of these single-copy homologous genes, which revealed the same phylogenetic relationship (Additional file 1: Fig. S4).

To explore the genomic diversity in Euplotia, we clustered the count of genes for each homologous group of Euplotia and Hypotrichia using the k-means algorithm. As shown in Fig. 2C, approximately 4159 homologous groups had no significant gain/loss in any species, and these may represent housekeeping genes in both subclasses. We enriched those genes in Gene Ontology database and found that most of them were involved in functions of catalytic activity, binding activity, metabolic process, or cellular process (Additional file 1: Fig. S5). Hypotrichia had 2822 homologous groups gained in the whole subclass. Euplotia, however, did not show a significant gain cluster at the subclass level; instead, seven clusters of homologous groups were found at the genus or species level. For example, *E. vannus* and *E. crassus* were more closely related to each other, sharing 1208 gained homologous groups, whereas *E. woodruffi*, *E. parawoodruffi*, and *E.* cf. *woodruffi* were clustered together and shared 2087 gained homologous groups. These findings indicated that Euplotia exhibited much higher genetic heterogeneity than Hypotrichia. To further quantify the genetic distances among several species of Euplotia and Hypotrichia, we used four representative species in each subclass and computed the sequence identities of their shared homolog groups (Fig. 2D). The median protein identity within Euplotia was approximately 35%, much lower than that of Hypotrichia (65%) (Fig. 2E). Taken together, these findings strongly indicate that the genomic diversity in Euplotia has been underestimated.

### Extensive gene expansion events in Euplotia

**Fig. 3:**
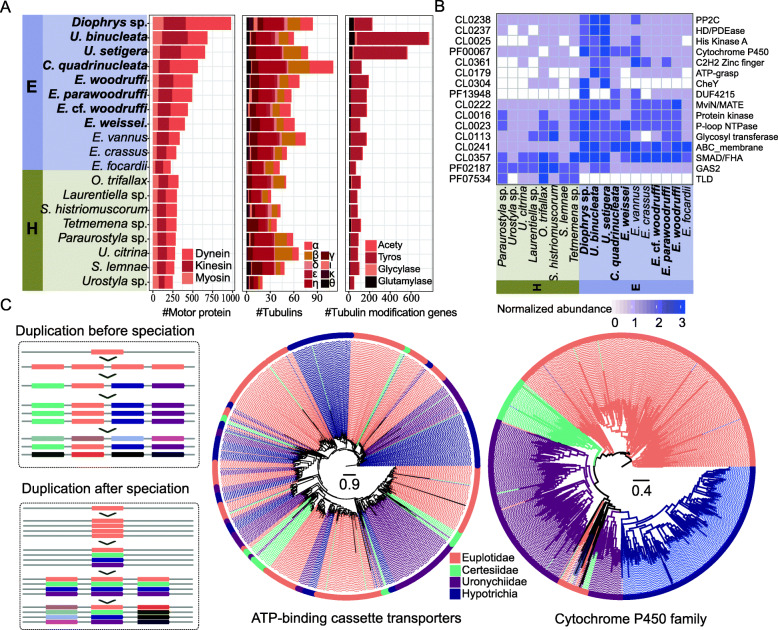
Extensive gene amplification events in Euplotia. **A** The counts of genes related to cilium kinetics in each species of Euplotia and Hypotrichia. **B** Significantly expanded/contracted gene families among all species of Euplotia and Hypotrichia. Each cell in the heatmap represents the normalized gene count of a Pfam family. **C** Gene duplication before and after speciation. The phylogenetic trees on the right side were constructed based on ABC transporters and Cytochrome P450 gene families using the ML method, respectively

To explore the gene expansion events of Euplotia genomes, we used both homology search approach and function prediction approach to classify genes of 11 Euplotia species and eight Hypotrichia species. We processed homology search approach using OrthoFinder and obtained 290,432 homologous groups. Of all the homologous groups, 1426 were presented in all species and seven were single-copy homologous groups. InterProScan [27] was used to identify conserved functional protein domains of genes in the 19 species. We finally obtained 5991 Pfam domains which were classified into 2897 clans. To investigate potential gene expansion events, we performed gene family gain/loss analysis to the 2897 clans using CAFE [28] and found that 234 clans showed significant gain/loss changes. The number of gain/loss genes was 628/411 in *Diophrys*, 596/281 in *Uronychia*, 365/1344 in *Certesia*, and 122/334 in *Euplotes* (Additional file 1: Fig. S6). By comparing Euplotia with Hypotrichia using Fisher’s exact test, 39 clans showed a significant discrepancy between the two subclasses (*P* < 0.01). Clans correlated to motor protein genes, tubulin genes, and tubulin modification genes were found to be significantly expanded in Euplotia (Fig. 3A). Motor protein genes and tubulin genes are closely associated with the ciliary movement in ciliates [29], which may imply Euplotia might have a better movement ability than Hypotrichia. In addition to genes related to ciliary movement, Euplotia species also underwent expansion in genes related to environmental adaptation, such as C2H2 zinc finger proteins, cytochrome P450, ATP-grasp gene, and ABC membrane (Fig. 3B).

To identify the relationship between gene expansion and speciation, we compared the results of homology search approach using OrthoFinder and function prediction approach using InterProScan. Among the 5991 annotated Pfam domains, 937 (15.6%) contain only one homologous group, and 585 (9.8%) contain more than five homologous groups. Among the 39 clans with a significant discrepancy between Euplotia and Hypotrichia, the cytochrome P450 clan contains 57 homologous groups and ATP-binding cassette transporters contain 42 homologous groups. To find out the relationship between these genes and speciation, we performed phylogenetic analysis of ABC transporter genes and cytochrome P450 genes. As shown in Fig. 3C, cytochrome P450 genes and ABC transporter represent two different modes of gene duplication. ABC transporter-related genes were divided into several subgroups, and genes from each species were dispersed in each group, indicating that most of these genes were amplified before speciation. In contrast, cytochrome P450 genes were classified into four subgroups consistent with the taxonomic classification, indicating that they may have been duplicated after speciation.

### Four distinct subgroups in genus *Euplotes*

**Fig. 4:**
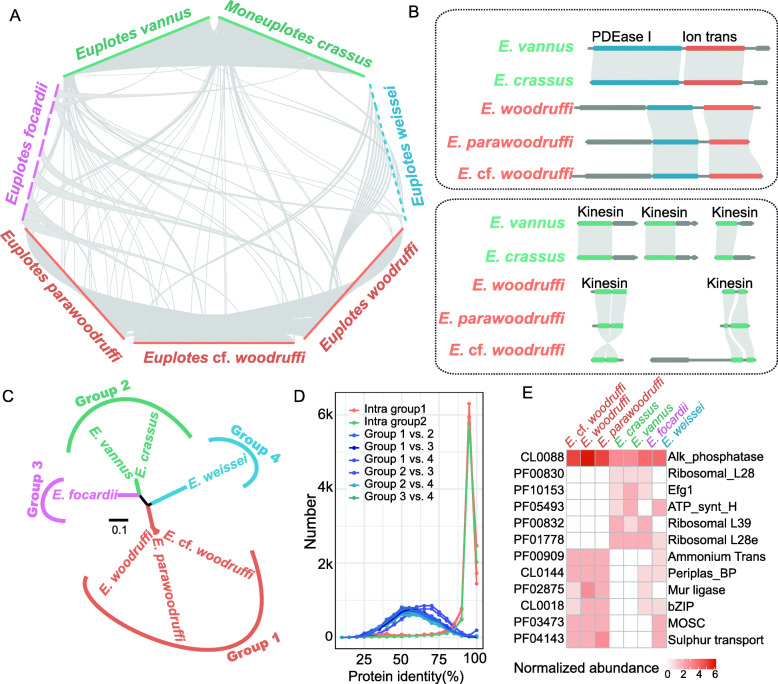
Genomic diversity of genus *Euplotes*. **A** Synteny analysis of the seven genomes in *Euplotes*. Only the contigs containing syntenic regions are showed in the figure. **B** Syntenic regions of cyclic nucleotide phosphodiesterase, ion transport protein, and kinesin. **C** Phylogenetic tree of the 157 orthologs found in *Euplotes* using the ML method. **D** The distribution of protein identities among the four *Euplotes* groups. **E** Significantly expanded/contracted gene families among all species of Euplotia and Hypotrichia

As the largest and most diverse genus of ciliates, *Euplotes* exhibits many genus-specific characteristics, such as unique genetic codons, a high frequency of programmed ribosomal frameshifts, well-developed AZMs, and a single undulating membrane [4, 21]. The genomic divergence of different species of *Euplotes* is still unknown because of the lack of genomic data. Here, the successful assembly of four new genomes in this genus enables a comprehensive comparison of genome structure and gene content among the different species. We first performed a synteny analysis of the seven *Euplotes* genomes and found that they could be divided into four subgroups based on their syntenic similarities (Fig. 4A). *E.* cf. *woodruffi*, *E. woodruffi*, and *E. parawoodruffi* formed one subgroup with an average of 512 syntenic regions. *E. vannus* and *M. crassus* formed another subgroup that shared 307 syntenic regions. The genomic syntenies of *E. focardii* and *E. weissei* were distinct from those of any other species and formed two separate subgroups.

We annotated genes in the same syntenic regions and found that these genes were generally functionally associated. For example, cyclic nucleotide phosphodiesterase (PDEase_I) and ion transporter (Ion_trans) were always adjacent to each other among the four subgroups despite their limited sequence identities (Fig. 4B). Previous studies found that cyclic nucleotide phosphodiesterase can regulate the activity of ion transport proteins by controlling cAMP or cGMP in cyclic nucleotide-gated ion channels [30]. Meanwhile, tandem or segmental duplication events (e.g., kinesin) may also contribute to the divergence of gene synteny between different subgroups (Fig. 4B).

To determine the phylogenetic relationships among the four subgroups, we first constructed a phylogenetic tree using 157 ortholog genes. The intrasubgroup phylogenetic distances were generally less than 0.01, which was significantly lower than those between subgroups (> 0.1) (*t* test, *P* < 0.01) (Fig. 4C). Then, we calculated the pairwise protein identity for each homologous group across different species. The number of shared homologous group and their protein identities within each subgroup were higher than those between subgroups. For example, subgroup 1 and subgroup 2 had 9785 and 8966 homologous genes, respectively, with an average amino acid identity > 95%. In contrast, *Euplotes* spp. had an average of 5663 shared genes between subgroups, with an average amino acid identity < 60% (Fig. 4D). We further used Fisher’s exact test to find significantly enriched or depleted Pfam domains among these subgroups. As shown in Fig. 4E, most of these Pfam domains/clans were involved in sulfur and ammonium metabolism and transport, which may be associated with environmental adaptations.

### Genomic characters of the newly sequenced genus *Uronychia*

*Uronychia* is a cosmopolitan ciliated organism that inhabits marine environments or saltwater. Although there are numerous studies concerning its morphology, morphogenesis, and taxonomy, the genus *Uronychia* remains one of the most mysterious ciliates due to the lack of whole-genome or transcriptome data [12, 31]. Compared to other species in Spirotricha, *Uronychia* has more rigid and sculptured body extracellular matrices, which makes its cell very stable. Meanwhile, *Uronychia* swims faster than other species and has rarely seen sudden jump behavior [2, 4, 32]. These characteristics indicate that *Uronychia* may have unique genome features related to cell wall generation and ciliary movement.

**Fig. 5:**
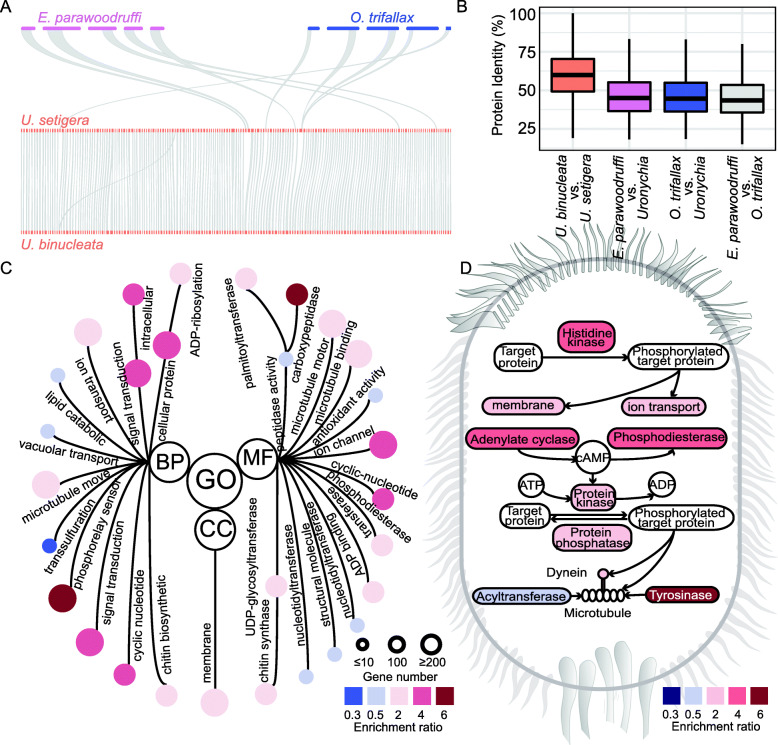
Genomic characteristics of genus *Uronychia*. **A** Synteny analysis between *Uronychia* spp*.* and their relatives, *E. parawoodruffi* and *O. trifallax*. Only the contigs containing syntenic regions are showed in the figure. **B** The distribution of protein identities between *Uronychia* spp*.* and their relatives. **C** Gene Ontology enrichment analysis in *Uronychia*. BP, CC, and MF represent the biological process, cellular component, and molecular function categories, respectively. The size of the circles represents the count of enriched genes, and the color of the circles represents the enrichment or depletion of functional terms in *Uronychia* compared to other species in Euplotia. **D** A schematic summary of ciliary movement machinery in *Uronychia*. The color of each component represents the enrichment or depletion of functional terms of *Uronychia* compared to other species in Euplotia

To explore the genetic divergence between the genus *Uronychia* and its relatives in Euplotia and Hypotrichia, we first compared the genomic synteny of two *Uronychia* species to the seven *Euplotes* and 10 Hypotrichia species we described above. The genomic structure between *U. setigera* and *U. binucleate was highly conserved* but divergent from that of other species. We further analyzed the genes shared in these species and their protein similarity. The number of homologous proteins and their protein identity between the two *Uronychia* is higher than that of other species. For example, U. setigera has 172 syntenic regions with U. binucleate, but only has five syntenic regions with E. parawoodruffi and five syntenic regions with *O. trifallax* (Fig. 5A). Meanwhile, protein identity of homologous genes between the two Uronychia species (63%) is higher than the average identity of *U. setigera*, *U. binucleate, E. parawoodruffi*, and *O. trifallax* (40%) (Fig. 5B).

Next, we performed Gene Ontology (GO) enrichment analysis for the two *Uronychia* species. Nine thousand eight hundred seventy-four genes of *U. binucleate* were classified into 711 GO terms, and 9838 genes of *U. setigera* were classified into 777 terms. We compared the enriched GO terms between *Uronychia* species and other 22 Spirotricha species using Fisher’s exact test and found 109 terms were significantly different (*P* < 0.01), including terms related to chitin synthesis, microtubule, signal transduction, sulfur metabolism, and carboxypeptidase. Chitin is an important component of ciliates [33], and there is a significant gene expansion of chitin synthesis gene in *Uronychia*, which is consistent with the morphological structure of *Uronychia*. Genes related to carboxypeptidase in *Uronychia* are six times higher than those in other Spirotricha species, indicating *Uronychia* may have diverse functions in protein degradation. Both adenylate cyclase genes and phospholipase genes were significantly expanded, which may suggest that *Uronychia* has a strong regulation of cyclic adenosine monophosphate (cAMP). In addition, genes related to transsulfuration, vacuolar transport, lipid catabolic, and antioxidant activity underwent contraction and genes related to ion transport and signal transduction underwent expansion (Fig. 5C).

We further proposed a model of ciliary movement machinery based on comparative genomic analysis (Fig. 5D). The axoneme, made up of nine doublet microtubules, is the scaffold of the cilia and provides tracks for molecular motors [34]. Dynein and kinesin on the axoneme move cilia through the intraflagellar transport process [35], in which ion transport proteins may provide energy for ciliary movement [36]. Cyclase and phosphodiesterase regulate the activity of ion transport proteins, dynein and the membrane by controlling the level of cAMP [37, 38], which is an important second messenger, to protein kinases by activating cilium-related proteins. As shown in Fig. 5D, *Uronychia* exhibited a significant expansion in adenylate cyclase, phosphodiesterase, protein kinase, and ion transporter, which may explain its unique and strong swimming ability.

## Discussion

As one of the most diverse ciliate subclass [4], the study of the genome diversity of Euplotia is important for understanding its phylogenetic history and environmental adaptation. Currently, only three species in this subclass have published genomes, and more genomic information from other genera is needed to study their genomic diversity. Although there have been some applications of single-cell sequencing in ciliates [9, 10, 39], the sequencing and analysis methods they used are not sufficient to obtain complete genomes, thus hindering the study of genomic diversity in ciliates. In this study, we optimized the single-cell sequencing methods and corresponding data analyses to effectively remove contamination by commensal bacteria and to generate high-quality ciliate genomes. We obtained eight ciliate genomes using this approach, and through comparative genomics analysis, we characterized the underestimated genomic diversity of Euplotia and determined the divergence time of representative species in this subclass for the first time; we also further investigated the extensive duplication events associated with speciation and environmental adaptation.

Single-cell sequencing has been applied to explore ciliate genomes and transcriptomes. Due to insufficient DNA content and high amplification bias, it is impossible to obtain a complete genome from a single cell. In addition, amplification of too many cells may increase the error rate caused by single-cell amplification and genomic differences between cells. By combining the advantages of MDA and MALBAC, we successfully obtained both better genome coverage and less amplification bias when processing the DNA samples of these uncultured ciliate cells. The incorporation of whole-genome sequence from a few more cells (2~4) can improve the final genome assembly without sacrificing homogeneity. In addition, excluding bacterial contamination is an important challenge in nonmodel organism genomics research. In this study, we found that the percentage of bacteria-derived reads in the raw sequencing data was generally below 10%, which was significantly lower than that in bulk whole-genome sequencing. Using the iterative filtration method, we were able to remove most of the nonciliate contaminants and obtain eight high-quality Euplotia genomes.

Through the combination of homology search approach and functional annotation approach, we found that genes related to environmental adaption exhibited huge expansion. When a gene duplication event occurs after speciation, the genes tend to vary greatly between species due to evolution. In contrast, when gene duplication events occur before speciation, the genes tend to divide into several lineages shared by all species (Fig. 3C). In highly expanded genes that are related to environmental adaption, we observed the relationship between gene duplication and speciation. This finding provides some insights into the correlation between speciation and environmental adaption. Through comparative analysis, we found that the diversity and complexity of Euplotia genomes were higher than expected. The genus *Euplotes* contains more than 80 species, most of which have similar cell shapes, cirri counts, and well-developed AZMs [4]. Based on an analysis of the assembled genomes from single-cell sequencing, we found that these seven species should be divided into four subgroups. In addition to the diversity of Euplotia, we further found that Euplotia exhibited chitin synthase gene expansion compared to Hypotrichia, which may be responsible for the more rigid lorica structure in Euplotia. This study demonstrates the power of single-cell genome sequencing in investigating the genomic diversity and novel characteristics of unculturable ciliates. The eight high-quality genomes obtained in this study will help understand the genetic basis of ciliate evolution and phenotypic adaptation.

## Conclusions

In this study, we optimized the single-cell sequencing methods and corresponding data analyses to effectively remove contamination by commensal bacteria and to generate high-quality ciliate genomes. We obtained eight ciliate genomes using this approach, and through comparative genomics analysis, we characterized the underestimated genomic diversity of Euplotia and determined the divergence time of representative species in this subclass for the first time. We further investigated the extensive duplication events associated with speciation and environmental adaptation. This study provides a unique and valuable resource for understanding the evolutionary history and genetic diversity of ciliates.

## Methods

### Sample collection and genome sequencing

*E. woodruffi* was collected from a freshwater pond in Taipingjiao Park in Qingdao, China. *E.* cf. *woodruffi* was collected from 17‰ brackish water pond in Shenzhen Binhai Park, China. *E. parawoodruffi* was collected from15‰ brackish water from Qingdao Coast, China. The other samples were collected from coastal seawater in Qingdao, China (Fig. 1B). All samples were poured into Petri dishes, and ciliate cells were isolated using glass pipettes. For microscopic inspection, the samples were silver stained to reveal their infraciliature. After morphological observation, the samples were washed 3–5 times to remove contaminants, and the genomic DNA was amplified using the REPLI-g Single Cell Kit (Qiagen) and the Single-Cell WGA Kit (Yikon, YK001A). For each species, two cells were subjected to each amplification method according to the manufacturer’s guidelines. Sequencing libraries were constructed using the TruSeq Nano DNA HT Sample Preparation Kit (Illumina). High-throughput sequencing was performed on an Illumina NovaSeq 6000 platform with PE150.

### Genome assembly

Sequencing data from the same species were evaluated using FastQC v0.11.9 (http://www.bioinformatics.babraham.ac.uk/projects/fastqc/) and merged. Fastp v0.20.1 [40] was used to trim adapters and polymers with default parameters. Low-quality reads were filtered with Trimmomatic 0.39 [41] (LEADING:20 TRAILING:20 SLIDINGWINDOW:4:25 MINLEN:120 AVGQUAL:28). Spades v3.13.0 [42] was used to assemble paired-end reads into contigs with single-cell option and multi-kmer options (-k 61, 71, 77, 81). Then, the following procedures were used to remove potential contamination: (1) MetaGeneMark v3.38 [43] was used to annotate assembled contigs; (2) gene fragments assigned to bacterial or algal species by searching against the NR database using DIAMOND v0.9.26 [44] were considered contaminants; and (3) Bowtie 2.4.1 [45] was used to align paired-end reads against the target bacterial genomes downloaded from the NCBI RefSeq database. Transcriptome datasets of Euplotia downloaded from the NCBI SRA database were assembled using Trinity 2.8.5 [46] with default parameters. QUAST v5.0.2 [47] with default parameters was used to determine the statistics of the assembled genomes. EukCC v0.2 [19] with default parameters was used to assess genome completeness at the protein level. BUSCO v4.0.6 [20] was used to assess the assembly quality using Alveolata dataset and protein mode.

### Gene prediction and annotation

AUGUSTUS v3.3.3 [48] was used to predict genes from assembled contigs. The eight species we sequenced used two nonstandard codons to translate proteins. The species *Certesia quadrinucleata*, *Diophrys* sp., *Uronychia binucleate*, and *Uronychia setigera* used “translation table 6” (https://www.ncbi.nlm.nih.gov/Taxonomy/Utils/wprintgc.cgi, TAA/TAG encodes glutamine compared to the standard code) as its codon, which is the same as *Tetrahymena*. The other four species, *Euplotes* cf. *woodruffi*, *Euplotes parawoodruffi*, *Euplotes weissei*, and *Euplotes woodruffi*, used “translation table 10” (TGA encodes cystine). For species using translation table 6, we used the *Tetrahymena* dataset to predict genes. For species using translation table 10, we modified the configuration file of the *Tetrahymena* dataset by choosing translation table 10, and then performed gene prediction. To ensure the quality of the predicted genes, we also trained AUGUSTUS by using nucleic acid and protein sequences of *Oxytricha trifallax* (http://oxy.ciliate.org/index.php/home/downloads) and *Euplotes vannus* (http://evan.ciliate.org/index.php/home/downloads). The NR database was used to annotate genes using DIAMOND v0.9.26 in protein search mode (*E* value < 1E−5). Motifs and protein domains were annotated against the PANTHER, Pfam, Gene3D, and CDD databases using InterProScan v10.0.2 [27]. GO and KEGG annotations for each gene were extracted from the corresponding InterPro entries.

### Gene family analysis

OrthoFinder v2.3.12 [26] was applied to find homologs among 24 Spirotricha species. In the OrthoFinder process, DIAMOND v0.9.26 was used to search sequence, MAFFT v7.470 [49] was used to align multiple sequences, and FastTree 1.0 [50] was used to infer phylogenetic tree. The homologous groups were clustered using k-means algorithm based on their gene abundance in each species. The protein identity for each homologous group between every species was calculated using a custom Perl script. All homologous groups were assigned to different Pfam domains based on the annotated results above. CAFE v4.2.1 [28] was used to compute gene expansion and contraction for the homologous groups between every species.

### Phylogenetic analysis and divergence time estimation

We used two different multiple alignment datasets for phylogenetic analysis. The first dataset contained single-copy genes of 24 Spirotricha species using protein by protein search process in OrthoFinder. The second dataset was obtained from GPSit v1.0 [22] pipeline by searching 157 orthologous genes in the 24 species. The sequences were trimmed using PREQUAL v1.02 [51] with threshold zero, aligned using MUSCLE v3.8.31 [52] with default parameters, retrimmed using Divvier v1.01 [53] with default parameters. After trimming and alignment, proteins of those two datasets were concatenated, respectively. BMGE v1.12 [54] was used to mask ambiguous sites of the alignments. IQ-Tree v1.6.12 [55] was used to construct the ML trees with C60+LG+G+F parameters. BEAST v1.10.4 [25] was used to construct the time-scaled trees under JTT substitution model and Strict clock model. Markov chain Monte Carlo (MCMC) sample chains were run for 10^7^. The convergence of parameters was evaluated using Tracer v1.7.1 [56].

### Genome synteny analysis

JCVI utility libraries v1.0.9 [57] were used to analyze and visualize the synteny between different genomes. LASTAL was used to perform pairwise synteny search with C-score cutoff of 0.7. MCScanX v1.0 [58] was used to detect syntenic regions with a cutoff of three genes. Genes in the same synteny region were extracted using custom Perl scripts and annotated using InterProScan v10.0.2 to determine the relationship between genome synteny and gene functions.

## Supplementary Information

**Additional file 1: Tables S1-S3, Figures S1-S6.** Supplementary tables and figures. **Table S1.** Single-cell amplification of eight Ciliates. Table on MDA and MALBAC methods for amplification of single cells. "1" means single-cell amplification success, "0" means amplification failure. For each amplification experiment, only one cell was used. **Table S2.** Information of 24 ciliate genomes. Table summarizing the data category, data source, accession number and hyperlink of the species used in this research. **Table S3.** Commands for analyzing process. Software, versions and parameters used for each analysis step. **Fig. S1.** Genome completeness assessed by BUSCO and EukCC. The completeness of the eleven Euplotia genomes assessed using EukCC and BUSCO software. Bold font indicates the species newly sequenced in this research, and regular font indicates species already published. **Fig. S2.** Percentage of contigs containing the number of genes. The proportion of contigs containing different gene counts out of the total contigs in each genome. Contigs containing more than 5 genes were grouped together. **Fig. S3.** Phylogenetic tree of all the sequenced ciliate species. Phylogenetic tree based on 157 gene sequences. The tree was constructed by the maximum likelihood method (C60+LG+G+F model). The numbers on the nodes represent the bootstrap values. **Fig. S4.** Phylogenetic tree based on single-copy genes. Phylogenetic tree of 19 Euplotia species base on single-copy genes obtained from compared genetic analysis. **Fig. S5.** Gene ontology enrichment. Gene ontology enrichment of housekeeping genes in Euplotia. The tree was constructed by the maximum likelihood method (C60+LG+G+F model). The numbers on the nodes represent the bootstrap values. **Fig. S6.** Gene gain/loss in this study. Gene gain/loss in Euplotia and Hypotrichia. Red letter represents gene gain, and blue letter represents gene loss.

## Data Availability

All data generated or analyzed during this study are included in this published article and its supplementary information files and publicly available repositories. Reads for the genome assemblies have been deposited in GenBank’s Short Read Archive (SRA) under BioProject number PRJNA721552. The software and parameters for each analysis step were listed in Additional file 1: Table S3. The single-gene alignments, the output of comparative genomics statistics, single copy homologous genes, and tables for drawing figures of this study are available at the Figshare website with DOI: 10.6084/m9.figshare.17078357. The custom scripts are available at the website Github: https://github.com/trainrun/Euplotes_script.
