# Supplementary material for: The hidden genomic diversity of ciliated protists revealed by single-cell genome sequencing
Source: BMC Biol. 2021 Dec 14;19:264. doi: 10.1186/s12915-021-01202-1 (PMC8670190; doi:10.1186/s12915-021-01202-1)
Supplement: Supplementary file 1 — Additional file 1: Tables S1-S3, Figures S1-S6. Supplementary tables and figures. Table S1. Single-cell amplification of eight Ciliates. Table on MDA and MALBAC methods for amplification of single cells. "1" means single-cell amplification success, "0" means amplification failure. For each amplification experiment, only one cell was used. Table S2. Information of 24 ciliate genomes. Table summarizing the data category, data source, accession number and hyperlink of the species used in this research. Table S3. Commands for analyzing process. Software, versions and parameters used for each analysis step. Fig. S1. Genome completeness assessed by BUSCO and EukCC. The completeness of the eleven Euplotia genomes assessed using EukCC and BUSCO software. Bold font indicates the species newly sequenced in this research, and regular font indicates species already published. Fig. S2. Percentage of contigs containing the number of genes. The proportion of contigs containing different gene counts out of the total contigs in each genome. Contigs containing more than 5 genes were grouped together. Fig. S3. Phylogenetic tree of all the sequenced ciliate species. Phylogenetic tree based on 157 gene sequences. The tree was constructed by the maximum likelihood method (C60+LG+G+F model). The numbers on the nodes represent the bootstrap values. Fig. S4. Phylogenetic tree based on single-copy genes. Phylogenetic tree of 19 Euplotia species base on single-copy genes obtained from compared genetic analysis. Fig. S5. Gene ontology enrichment. Gene ontology enrichment of housekeeping genes in Euplotia. The tree was constructed by the maximum likelihood method (C60+LG+G+F model). The numbers on the nodes represent the bootstrap values. Fig. S6. Gene gain/loss in this study. Gene gain/loss in Euplotia and Hypotrichia. Red letter represents gene gain, and blue letter represents gene loss. [file 12915_2021_1202_MOESM1_ESM.docx]

**Table S1. Single-cell amplification of eight Ciliates.**

| Species | MDA | | MALBAC | |
| --- | --- | --- | --- | --- |
| *Certesia quadrinucleata* | 1 | 1 | 1 | 1 |
| *Diophrys* sp. | 1 | 1 | 1 | 0 |
| *Euplotes* cf. *woodruffi* | 1 | 0 | 1 | 1 |
| *Euplotes parawoodruffi* | 1 | 0 | 1 | 1 |
| *Euplotes weissei* | 1 | 1 | 1 | 1 |
| *Euplotes woodruffi* | 1 | 1 | 1 | 1 |
| *Uronychia binucleata* | 0 | 0 | 1 | 0 |
| *Uronychia setigera* | 1 | 1 | 1 | 1 |

| **Table S2. Information of 24 ciliate genomes.** | | | | | |
| --- | --- | --- | --- | --- | --- |
|  |  |  |  |  |  |
| **Species** | **Data category** | **source** | **Accession number** | **Website** | **Number of genes** |
| *Favella ehrenbergii* | transcriptome | NCBI | SRR1296812 | https://www.ncbi.nlm.nih.gov/ | 14,142 |
| *Schmidingerella taraikaensis* | transcriptome | NCBI | SRR1296899 | https://www.ncbi.nlm.nih.gov/ | 12,962 |
| ***Certesia quadrinucleata*** | genome | *This study* | *to be released* | https://www.ncbi.nlm.nih.gov/ | 15,982 |
| ***Diophrys* sp.** | genome | *This study* | *to be released* | https://www.ncbi.nlm.nih.gov/ | 25,327 |
| ***Euplotes* cf. *woodruffi*** | genome | *This study* | *to be released* | https://www.ncbi.nlm.nih.gov/ | 14,298 |
| *Moneuplotes crassus* | genome | NCBI | GCA_001880385.1 | https://www.ncbi.nlm.nih.gov/ | 11,481 |
| *Euplotes focardii* | genome | NCBI | GCA_001880345.1 | https://www.ncbi.nlm.nih.gov/ | 9,382 |
| ***Euplotes parawoodruffi*** | genome | *This study* | *to be released* | https://www.ncbi.nlm.nih.gov/ | 19,635 |
| *Euplotes vannus* | genome | ciliates.org | - | http://ciliates.org/ | 15,078 |
| ***Euplotes woodruffi*** | genome | *This study* | *to be released* | https://www.ncbi.nlm.nih.gov/ | 15,830 |
| ***Euplotes weissei*** | genome | *This study* | *to be released* | https://www.ncbi.nlm.nih.gov/ | 21,295 |
| ***Uronychia binucleata*** | genome | *This study* | *to be released* | https://www.ncbi.nlm.nih.gov/ | 19,733 |
| ***Uronychia setigera*** | genome | *This study* | *to be released* | https://www.ncbi.nlm.nih.gov/ | 20,152 |
| *Halteria grandinella* | genome | NCBI | GCA_006369765.1 | https://www.ncbi.nlm.nih.gov/ | 27,152 |
| *Laurentiella* sp. | genome | NCBI | GCA_001272975.2 | https://www.ncbi.nlm.nih.gov/ | 13,594 |
| *Oxytricha trifallax* | genome | ciliates.org | - | http://ciliates.org/ | 20,136 |
| *Pseudokeronopsis carnea* | genome | NCBI | GCA_006510595.1 | https://www.ncbi.nlm.nih.gov/ | 29,520 |
| *Paraurostyla* sp. | genome | NCBI | GCA_001272965.2 | https://www.ncbi.nlm.nih.gov/ | 14,448 |
| *Sterkiella histriomuscorum* | genome | NCBI | GCA_001273305.2 | https://www.ncbi.nlm.nih.gov/ | 19,845 |
| *Stylonychia lemnae* | genome | NCBI | GCA_000751175.1 | https://www.ncbi.nlm.nih.gov/ | 16,786 |
| *Tetmemena* sp. | genome | NCBI | GCA_001273295.2 | https://www.ncbi.nlm.nih.gov/ | 20,551 |
| *Uroleptopsis citrina* | genome | NCBI | GCA_001653735.1 | https://www.ncbi.nlm.nih.gov/ | 14,395 |
| *Urostyla* sp. | genome | NCBI | GCA_001272955.2 | https://www.ncbi.nlm.nih.gov/ | 8,617 |
| *Strombidium stylifer* | genome | NCBI | GCA_010577775.1 | https://www.ncbi.nlm.nih.gov/ | 24,057 |

**Table S3. Commands for analyzing process.**

| Reads filter and quality control | java -jar trimmomatic-0.39.jar PE -threads 12 -phred33 fastq1 fastq2 out1 out2 out3 out4 ILLUMINACLIP:TruSeq3-PE.fa:2:30:10 |
| --- | --- |
|  | java -jar trimmomatic-0.39.jar PE -threads 12 -phred33 fastq1 fastq2 out1 out2 out3 out4 LEADING:20 TRAILING:20 SLIDINGWINDOW:4:25 MINLEN:120 AVGQUAL:28 |
|  | fastqc -t 12 -o qc_dir fastq_file |
| Genome assmeble and pollution filter | spades.py -t 12 --sc -k 61,71,77,81 -o output_dir -1 fastq1 -2 fastq2 |
|  | gmhmmp -a -f G -m MetaGeneMark_v1.mod -A protein.faa scaffolds.fasta |
|  | diamond blastp --db nr.dmnd --outfmt 100 --threads 6 --query protein.faa --out output.blast --outfmt 6 qseqid sseqid pident length mismatch gapopen qstart qend sstart send evalue bitscore stitle |
|  | bowtie2 -x ref -1 fastq1 -2 fastq2 -p 12 --very-sensitive-local -X 2000 --un-conc unmap |
| Gene prediction and annotation | optimize_augustus.pl --species=oxytricha --cpus=12 Oxytricha_genomic.gbff.train |
|  | augustus --genemodel=complete --protein=on --introns=on --start=on --stop=on --cds=on --codingseq=on --gff3=on --outfile=out.gff --species=tetrahymena file_name |
|  | augustus --genemodel=complete --protein=on --introns=on --start=on --stop=on --cds=on --codingseq=on --gff3=on --outfile=out.gff --species=euplotes file_name |
|  | diamond blastp --db nr.dmnd --threads 12 --query faa --out out.blast --outfmt 6 qseqid sseqid pident length mismatch gapopen qstart qend sstart send evalue bitscore stitle |
| Assembly evalutation | quast.py -t 10 scaffolds.fasta -o quast_dir |
|  | eukcc -n 12 -o outdir --plot --db eukccdb --proteins prot.faa |
|  | busco -i fasta -l alveolata_odb10 -o out_dir -m prot --cpu 12 |
| Phylogenetics | perl GPSit.pl -i faa_file -n ref_file -o output_dir -e 1e-10 -d 50 -g 100000 -f 100 -t 15 |
|  | prequal faa_file |
|  | muscle -in faa_file -out out_file |
|  | divvier -mincol 4 -divvygap alignment_file |
|  | java -jar BMGE.jar -i alignment_file -t AA -g 0.3 -b 5 -of masked_file -oh html |
|  | iqtree -s masked_file -m LG+C60+F+G -bb 1000 -ntmax 12 |
| Homology analysis | orthofinder -f folder -a 12 -t 12 |
| Annotation | interproscan.sh -i faa_file -d out_dir -dp -cpu 12 -pa -iprlookup -goterms -appl Pfam |
| Synteny analysis | python -m jcvi.compara.catalog ortholog name1 name2 --no_strip_names -n 3 |
|  | python -m jcvi.compara.synteny screen --simple 1.2.anchors 1.2.anchors.new |
|  | python -m jcvi.graphics.karyotype seqids layout |


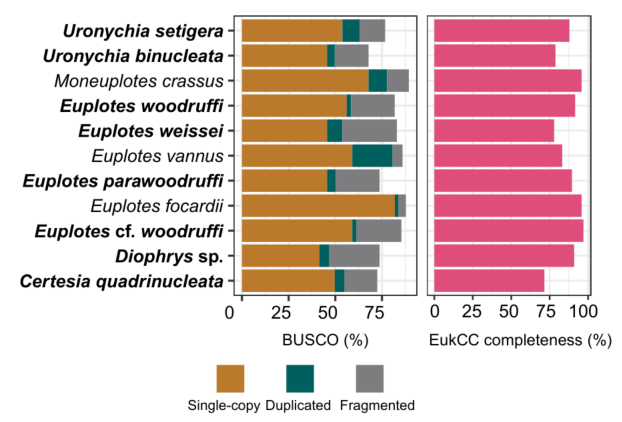


**Figure S1. Genome completeness assessed by BUSCO and EukCC.**


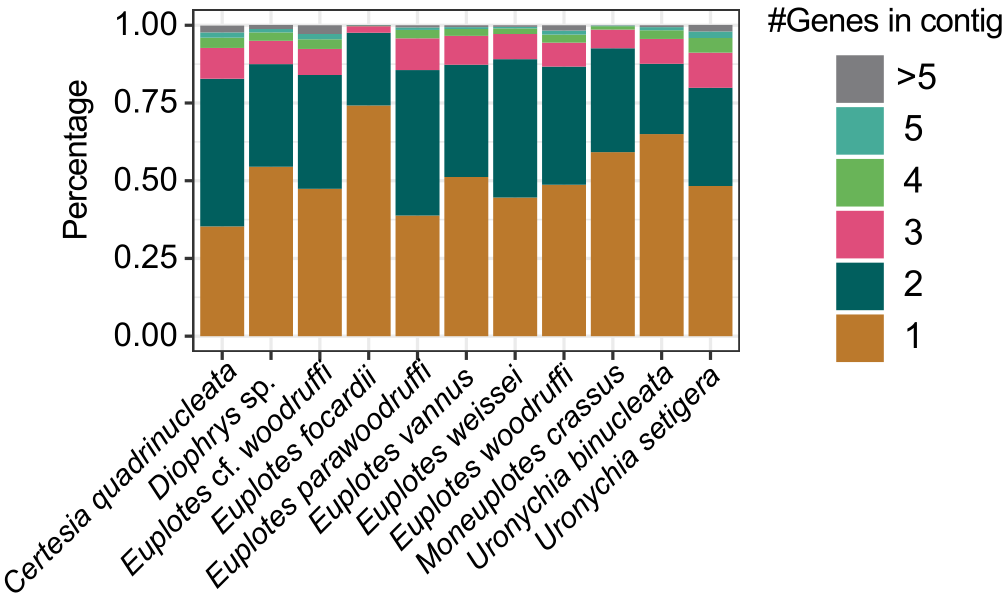


**Figure S2. Percentage of contigs containing the number of genes.**


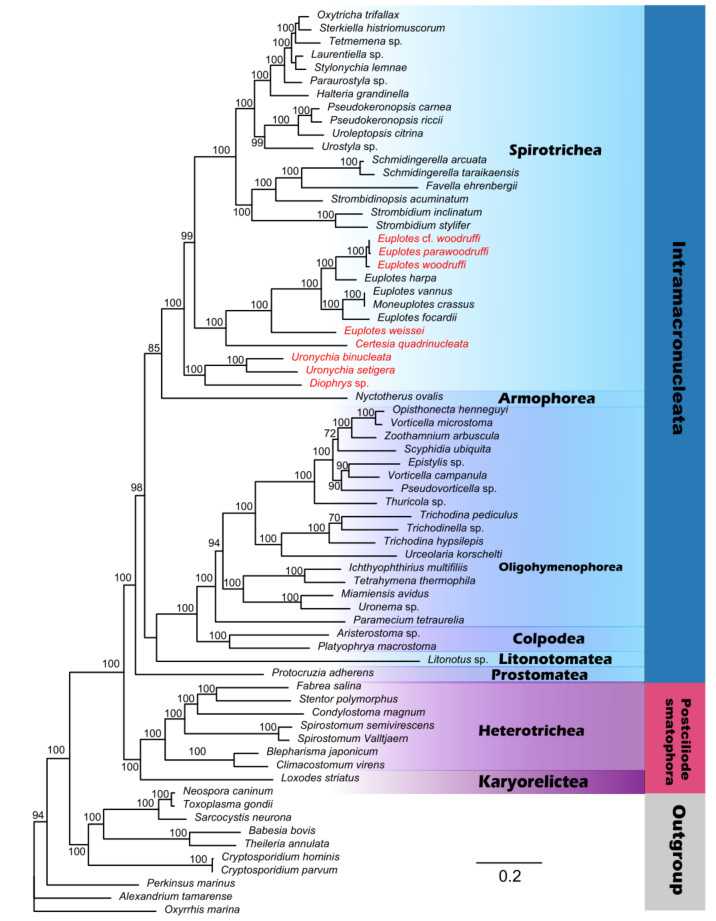


**Figure S3. Phylogenetic tree of all the sequenced ciliate species.**


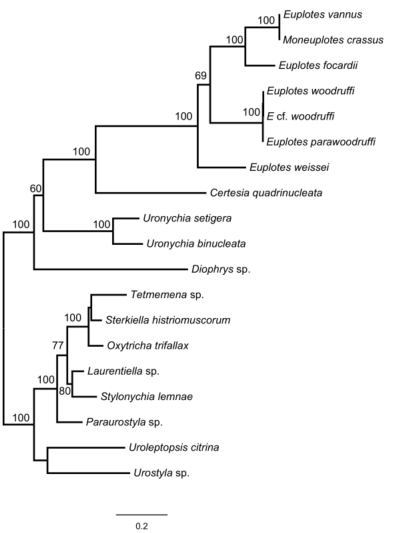


**Figure S4. Phylogenetic tree based on single-copy genes.**


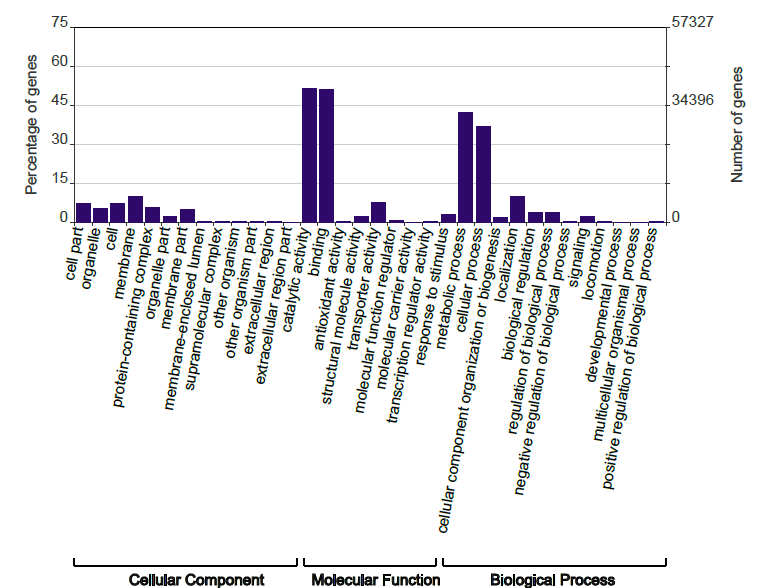


**Figure S5. Gene ontology enrichment.**


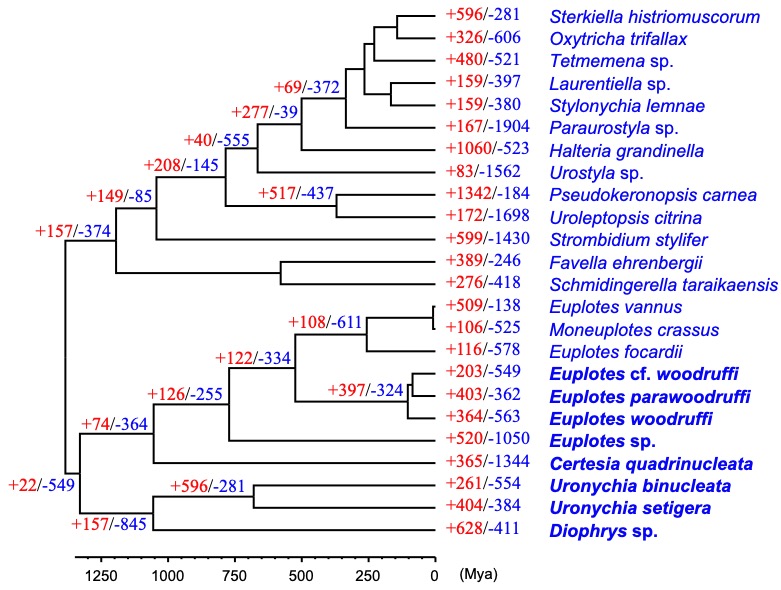


**Figure S6. Gene gain/loss in this study.**
